# Electron Correlation in the Iron(II) Porphyrin by Natural Orbital Functional Approximations

**DOI:** 10.1021/acs.jctc.2c01093

**Published:** 2022-12-29

**Authors:** Juan Felipe Huan Lew-Yee, Jorge M. del Campo, Mario Piris

**Affiliations:** † Departamento de Física y Química Teórica, Facultad de Química, Universidad Nacional Autónoma de México, México CityC.P. 04510, México; ‡ Kimika Fakultatea, Euskal Herriko Unibertsitatea (UPV/EHU), P.K. 1072, 20080Donostia, Euskadi, Spain; ¶ Donostia International Physics Center (DIPC), 20018Donostia, Euskadi, Spain; § IKERBASQUE, Basque Foundation for Science, 48013Bilbao, Euskadi, Spain

## Abstract

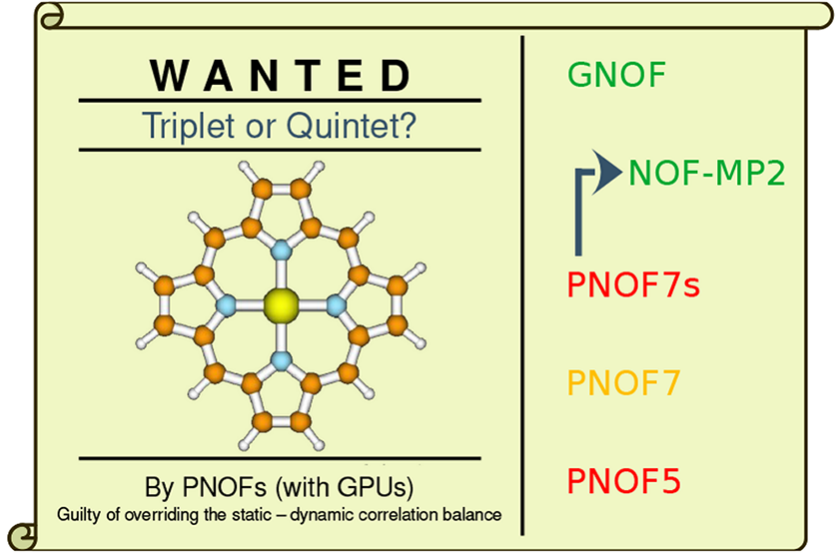

The relative stability of the singlet, triplet, and quintet spin states of iron(II) porphyrin (FeP) represents a challenging problem for electronic structure methods. While it is currently accepted that the ground state is a triplet, multiconfigurational wave function-based methods predict a quintet, and density functional approximations vary between triplet and quintet states, leading to a prediction that highly depends on the features of the method employed. The recently proposed Global Natural Orbital Functional (GNOF) aims to provide a balanced treatment between static and dynamic correlation, and together with the previous Piris Natural Orbital Functionals (PNOFs), allowed us to explore the importance of each type of correlation in the stability order of the states of FeP with a method that conserves the spin of the system. It is noteworthy that GNOF correlates all electrons in all available orbitals for a given basis set; in the case of the FeP with a double-ζ basis set as used in this work, this means that GNOF can properly correlate 186 electrons in 465 orbitals, significantly increasing the sizes of systems amenable to multiconfigurational treatment. Results show that PNOF5, PNOF7s, and PNOF7 predict the quintet to have a lower energy than the triplet state; however, the addition of dynamic correlation via second-order Møller–Plesset corrections (NOF-MP2) turns the triplet state to be lower than the quintet state, a prediction also reproduced by GNOF that incorporates much more dynamic correlation than its predecessors.

## 1 Introduction

As early as the 1970s, it was suggested that one-particle reduced density matrix (RDM) functional theory^1−4^ could be an attractive alternative formalism to wave function-based methods. Unfortunately, calculations based on exact functionals generated by the constrained-search formulation are computationally too expensive, which has prompted the development of approximate functionals for practical applications. The functionals currently in use are constructed on the basis where the one-particle RDM is diagonal, which is the definition of a natural orbital functional (NOF).^5,6^ In fact, it is more appropriate to speak of a NOF rather than a one-particle RDM functional when dealing with approximate functionals, since a two-particle RDM dependence persists^7^ and leads to the functional N-representability problem.^8,9^ An extensive account on the evolution of approximate NOFs up to the year 2018 can be found elsewhere.^10−13^

Recent developments^14−47^ show that NOF theory has become an active field of research. Nowadays, an open-source implementation of NOF-based methods is available (github.com/DoNOF) to the scientific community. The associated computer program DoNOF (Donostia Natural Orbital Functional)^48^ is designed to solve the energy minimization problem of an approximate NOF, describing the ground state of an N-electron system in terms of natural orbitals (NOs) and their occupation numbers (ONs). Fractional occupancies naturally allow NOFs to recover the static correlation. In fact, approximate NOFs have demonstrated^41,49^ to be more accurate than their electron density-dependent counterparts for highly multiconfigurational systems and scale satisfactorily compared to wave function-type methods with respect to the number of basis functions.

Particularly successful in describing static electronic correlation are electron-pairing-based NOFs,^50^ namely, PNOF5,^51,52^ PNOF6,^53^ and PNOF7.^54,55^ For instance, the PNOF6 dissociation curve of the carbon dimer closely resembles that obtained from the optimized complete active space self-consistent field wave function.^56^ So far, only NOFs that satisfy the electron-pairing constraints have provided the correct number of electrons in the fragments after homolytic dissociation.^57,58^ PNOF5–PNOF7 take into account most of the nondynamical effects, and also an important part of the dynamic electron correlation corresponding to the intrapair interactions; hence they produce results that are in good agreement with accurate wave function-based methods for small systems, where electron correlation effects are almost entirely intrapair. However, when the number of pairs increases, the total energy values deteriorate, especially in those regions where dynamic correlation prevails.

There are several strategies for adding the missing dynamic correlation to an approximate NOF, but second-order perturbative corrections are probably the simplest and cheapest way to properly incorporate dynamical correlation effects, which has given rise to two methods. The first uses a size-consistent multiconfigurational second-order perturbation theory (PT2), taking as reference the generating wave function of PNOF5, which leads to the PNOF5-PT2 method.^59,60^ The other proposal, called NOF-MP2,^54^ adds second-order Møller–Plesset (MP2) corrections to a reference Slater determinant wave function formed with the NOs of PNOF7. Let us note that PNOF5 is strictly N-representable; i.e., the functional can be derived from a wave function that is antisymmetric in N-particles, so PNOF5-PT2 is well-defined and the perturbative corrections are added to PNOF5 energy. On the contrary, for PNOF7 the generating wave function is unknown, and in the NOF-MP2 method static and dynamic corrections are added to a Hartree–Fock (HF) type energy.

The reformulation^61^ of NOF-MP2 based on the static part of PNOF7 (PNOF7s) and the orbital-invariant MP2 allowed us to prevent reference ONs and NOs from being spuriously influenced by nondynamic correlation in dynamic correlation domains and extend the NOF-MP2 method to any type of orbitals, including localized ones, respectively. NOF-MP2 has been shown to provide quantitative agreement for dissociation energies, with performance comparable to that of the accurate complete active space second-order perturbation theory in hydrogen abstraction reactions,^19^ and is highly reliable for accurate chemical reaction mechanistic studies in elementary reactions of transition metal compounds.^31^

A canonicalization procedure applied to the NOs gave us the possibility to combine any many-body perturbation method,^62^ like random-phase approximation or coupled-cluster singles and doubles, with a NOF. The inclusion of perturbative corrections improves the absolute energies over the reference NOF values and approaches the energies obtained by accurate wave function-based methods; however, it does not improve the quality of the reference NOs and ONs. A full optimization would be the only way to obtain completely correlated ONs and NOs. Unfortunately, such a self-consistent procedure makes perturbative methods incredibly computationally expensive, so it is preferable to recover the missing dynamic correlation using a more general NOF than PNOF7.

An important recent development that reinforced this strategy was the implementation of the resolution of the identity approximation (RI) in DoNOF^30^ and in the FermiONs++ program package.^40^ The RI implementation substantially reduces memory and arithmetic scaling factors in NOF calculations. Such developments have made it possible to perform calculations on large systems of chemical interest with tens of atoms, hundreds of electrons, and thousands of basis functions, for example, the 117-atom 2′-carbamate taxol and the 168-atom valinomycin molecule.^40^

Recently,^38^ a NOF was proposed for electronic systems with any spin value regardless of the external potential, that is, a global NOF (GNOF). The adjective “global” is used instead of “universal” to differentiate this approximate multipurpose NOF from Valone’s exact one.^4^ GNOF is able to achieve a balanced treatment of static and dynamic electron correlations even for those systems with significant multiconfigurational character, preserving the total spin of multiplets.^14^ It should be noted that the agreement obtained by GNOF with accurate wave function-based methods is not only for relative energies but also for absolute energies, a sign of good results for good reasons. An example is the agreement obtained between GNOF and Full Configuration Interaction (FCI) for challenging dissociation processes in one, two, and three dimensions.^41^ Nevertheless, we must point out that GNOF, like its predecessors, is not variational since only some necessary N-representability conditions have been imposed, with the sole exception of PNOF5 for which we know the generating wave function.

**Figure 1:**
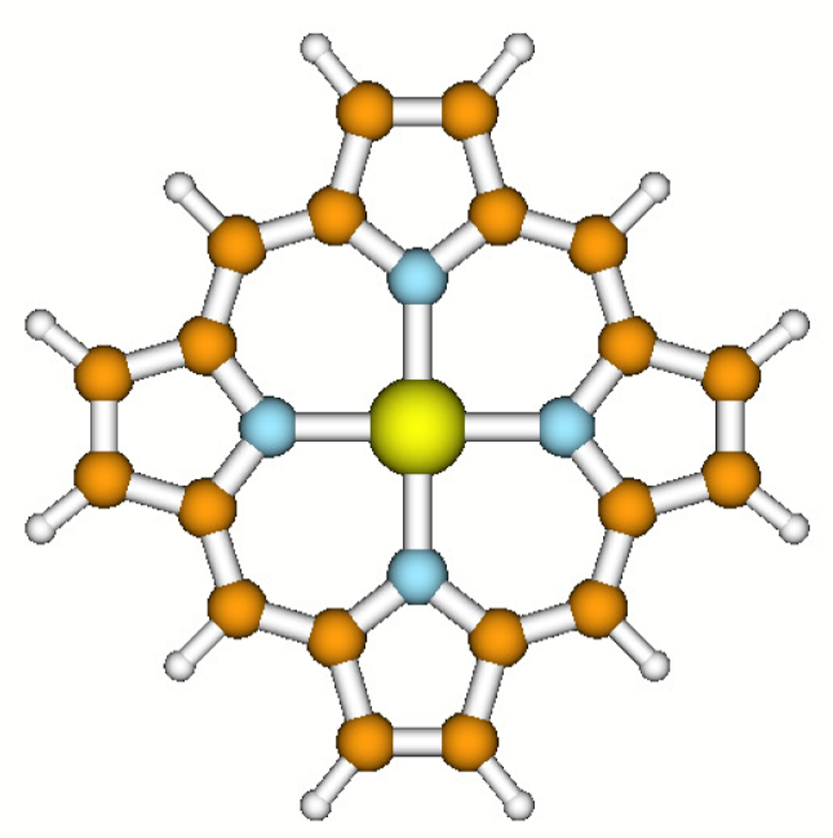
Iron(II) Porphyrin.

The simple construction of GNOF allowed us to examine the effects of different types of electron correlations. The functional has a term that fully recovers the intrapair electron correlation, that corresponds to the independent-pair model, followed by a second term that corresponds to the static interpair correlation, and it also takes into account the dynamic correlation between electron pairs. The aim of this work is to analyze the influence of different types of correlation on the spin state stability of iron(II) porphyrin molecule (FeP), as shown in Figure 1, a system with 37 atoms and 186 electrons. FeP is a model system for more general substituted iron porphyrins that play a vital role in many biological processes, including oxygen transport, electron transfer, and catalyzing the incorporation of oxygen into other molecules.^63^ The relationship between spin state and structure of FeP constitutes an active research topic^64^ due to its implications for the biological activity of heme proteins.^65,66^

The iron porphyrins have proven to be challenging for any theoretical method and an attractive system for testing the GNOF. Initial single reference studies considered a triplet state,^67−70^ but subsequent multireference studies favored a quintet state.^71,72^ The controversy about the spin of the ground state of FeP continues to this day, and the discussion has become enriched with the increase of computational power and the development of innovative methods to include electronic correlation more accurately.

Calculations with currently used single reference methods such as coupled cluster and modern density functional approximations tend to favor the triplet as the ground state,^73−75^ and the triplet and quintet states have been reported to not present essential symmetry breaking.^76^ In addition, typical complete active space (CAS) calculations point to the quintet,^77^ but increasing the size of the active space changes the prediction to the triplet; it has been stated that the preference for a quintet may be an artifact caused by an insufficiently large active space.^78,79^ Pair density functional theory (PDFT) points to the triplet,^80^ as well as calculations of stochastic generalized active space self-consistent field.^81^ However, the discussion is not so easy to conclude, as the density matrix renormalization group (DMRG) points to a quintet state,^82^ even after coupling with the adiabatic connection to include dynamic correlation.^83^ Studies on the influence of the exact exchange on DFAs concluded that the inclusion of a large amount of it favors high-spin states, while smaller contributions favor low-spin states,^84,85^ and this becomes relevant as a recent study of PDFT has found that the use of hybrid functionals reverts the tendency to the quintet state for some on-top functional.^86^ Hence, the controversy remains of active interest.

This study provides important information in many ways. First, the analysis of FeP from the perspective of PNOF functionals might provide information on the static and dynamic correlation effects on the problem. At the same time, it will allow us to compare the set of PNOFs with the different methods previously used to study FeP. Note further that GNOF correlates all electrons into all available orbitals for a given basis set, which in the case of FeP using a double-ζ basis set correlates 186 electrons in 465 orbitals. To the best of our knowledge, such a correlation calculation is not possible with current wave function-based methods, such as CAS or DMRG.

The work is organized as follows. First, Section 2 presents a brief review of GNOF and the M diagnostic used to characterize the NOFs solutions. This is followed by the computational details related to the NOF calculations in Section 3. Section 4 presents an analysis of the performance of PNOF5, PNOF7, PNOF7s, NOF-MP2, and GNOF over the spin-stability order of FeP, together with a discussion of the electron correlation effects provided by each functional. Finally, conclusions are given in Section 5.

## 2 Theory

In this section, we briefly describe GNOF, and a more detailed description can be found in ref (38). The nonrelativistic Hamiltonian under consideration is spin coordinate free; therefore, a state with total spin *S* is a multiplet, i.e., a mixed quantum state that allows all possible *S*_*z*_ values. We consider N_I_ single electrons which determine the spin *S* of the system, and the rest of electrons (N_II_ = N – N_I_) are spin-paired so that all spins corresponding to N_II_ electrons altogether provide a zero spin. In the absence of single electrons (N_I_ = 0), the energy obviously reduces to a NOF that describes singlet states.

We focus on the mixed state of highest multiplicity: 2*S* + 1 = N_I_ + 1, *S* = N_I_/2.^14^ For an ensemble of pure states , we note that the expected value of  for the whole ensemble is zero. Consequently, the spin-restricted theory can be adopted even if the total spin of the system is not zero. We use a single set of orbitals for α and β spins. All the spatial orbitals will be then doubly occupied in the ensemble so that occupancies for particles with α and β spins are equal: *n*_*p*_^α^ = *n*_*p*_^β^ = *n*_*p*_.

We divide the orbital space Ω into two subspaces: Ω = Ω_I_ ⊕ Ω_II_. Ω_II_ is composed of N_II_/2 mutually disjoint subspaces Ω_*g*_. Each of which contains one orbital |*g*⟩ with *g* ≤ N_II_/2 and N_*g*_ orbitals |*p*⟩ with *p* > N_II_/2, namely,
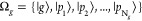
1Taking into account the spin, the total occupancy for a given subspace Ω_*g*_ is 2, which is reflected in the following sum rule:
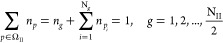
2

Here, the notation *p* ∈ Ω_II_ represents all the indexes of |*p*⟩ orbitals belonging to Ω_II_. In general, N_*g*_ can be different for each subspace as long as it describes the electron pair well. For convenience, in this work we take it to be equal for all subspaces Ω_*g*_ ∈ Ω_II_ to the maximum possible value determined by the basis set used in calculations. From eq 2, it follows that
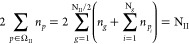
3Similarly, Ω_I_ is composed of N_I_ mutually disjoint subspaces Ω_*g*_. In contrast to Ω_II_, each subspace Ω_*g*_ ∈ Ω_I_ contains only one orbital *g* with 2*n*_*g*_ = 1. It is worth noting that each orbital is completely occupied individually, but we do not know whether the electron has α or β spin: *n*_*g*_^α^ = *n*_*g*_^β^ = *n*_*g*_ = 1/2. It follows that
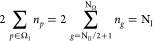
4In eq 4, N_Ω_ = N_II_/2 + N_I_ denotes the total number of suspaces in Ω. Taking into account eqs 3 and 4, the trace of the 1RDM is verified equal to the number of electrons:
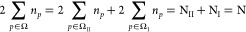
5Using ensemble N-representability conditions, we can generate a reconstruction functional for the 2RDM in terms of the ONs that leads to GNOF:
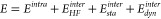
6The intrapair component is formed by the sum of the energies of the pairs of electrons with opposite spins and the single-electron energies of the unpaired electrons, namely,
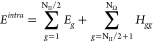
7
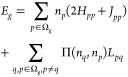
8where
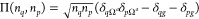
9and *H*_*pp*_ are the diagonal one-electron matrix elements of the kinetic energy and external potential operators. *J*_*pq*_ = ⟨*pq*|*pq*⟩ and *L*_*pq*_ = ⟨*pp*|*qq*⟩ are the Coulomb and exchange-time-inversion integrals, respectively. Ω^*a*^ denotes the subspace composed of orbitals above the level N_Ω_ (*p* > N_Ω_). The intersubspace HF term is
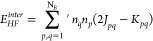
10where *K*_*pq*_ = ⟨*pq*|*qp*⟩ are the exchange integrals. The prime in the summation indicates that only the intersubspace terms are taking into account (*p* ∈ Ω_*f*_, *q* ∈ Ω_*g*_, *f* ≠ *g*). N_*B*_ represents the number of basis functions considered. The intersubspace static component is written as
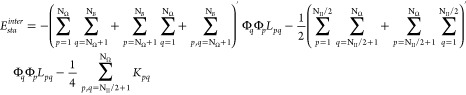
11where  with the hole *h*_*p*_ = 1 – *n*_*p*_. Note that Φ_*p*_ has significant values only when the occupation number *n*_*p*_ differs substantially from 1 and 0. Finally, the intersubspace dynamic energy can be conveniently expressed as
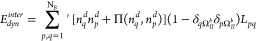
12

In eq 12, Ω_*II*_^*b*^ denotes the subspace composed of orbitals below the level N_II_/2 (*p* ≤ N_II_/2), so interactions between orbitals belonging to Ω_*II*_^*b*^ are excluded from *E*_*dyn*_^*inter*^. The dynamic part of the ON *n*_*p*_ is defined as
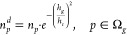
13with .^38^ The maximum value of *n*_*p*_^*d*^ is around 0.012 in accordance with the Pulay’s criterion that establishes an occupancy deviation of approximately 0.01 with respect to 1 or 0 for a NO to contribute to the dynamic correlation. Clearly, GNOF does not take into account the dynamic correlation of single electrons (*p* ∈ Ω_I_) via the *E*_*dyn*_^*inter*^ term. Considering real spatial orbitals (*L*_*pq*_ = *K*_*pq*_) and *n*_*p*_ ≈ *n*_*p*_^*d*^, it is not difficult to verify that the terms proportional to the product of the ONs will cancel out, so that only those terms proportional to Π will contribute significantly to the energy.

It is important to note that GNOF preserves the total spin of the multiplet: .^14^ Taking into account that GNOF does not contain intersubspace terms between orbitals below N_B_, except for the HF-like terms of the eq 10, eq 6 reduces to the PNOF7-like functional^54,55^ when the interpair dynamic term (*E*_*dyn*_^*inter*^) is neglected. Furthermore, taking Φ_*p*_ = 2*n*_*p*_*h*_*p*_ in eq 11 the PNOF7s-like version of the functional is obtained.^61^ Finally, if the intersubspace static term (*E*_*sta*_^*inter*^) is also disregarded, then GNOF reduces to PNOF5.^52^

The solutions of PNOFs can be characterized according to the recently proposed M-diagnostic^87^ adapted to the NOF multiplet calculations,^47^ namely,
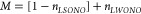
14where LSONO stands for the least strongly occupied NO, that is, the orbital with ON farthest from 1 below N_II_/2, so it belongs to Ω_*II*_^*b*^ subspace, and LWONO for the least weakly occupied NO, that is, the orbital with ON farthest from 0 above *N*_Ω_, so it belongs to Ω^*a*^ subspace. Recall that M values close to zero indicate the predominance of dynamic correlation, while values beyond 0.1 indicate the predominance of static correlation.

## 3 Computational Details

In this work, we have used the optimized structures of FeP reported in ref (74) for the singlet, triplet, and quintet states, as has been used in subsequent studies;^40,76,88^ hence the energy gaps are computed adiabatically. The Fe–N distance might be relevant for the energetics of the problem; in the used structures this distance corresponds to 1.979 Å for the singlet, 1.976 Å for the triplet, and 2.053 Å for the quintet. The solution of the NOF equations has been established by optimizing the energy separately with respect to the ONs and to the NOs. Therefore, orbitals vary along the optimization process until the most favorable orbital interactions are found. NOF-MP2 calculations have been carried out as described in ref (62). We have taken this opportunity to test an in-house piece of software written in Julia, currently named DoNOF.jl, and with integral transformation accelerated by graphic processing units (GPUs) in the calculations of the perfect pairing approach (N_g_ = 1), while the extended pairing calculations (N_g_ = 4) have been carried out using the DoNOF code.^48^ The correlation-consistent valence double-basis set including polarization (cc-pVDZ)^89,90^ was used throughout, as has been previously reported that the active space is more important than using a larger basis set (e.g., cc-pVTZ) to achieve the correct prediction.^40,78^ The resolution of the identity (RI) was used to reduce the computational cost of the calculations, as reported in ref (91) (including for NOF-MP2), and the cc-pVDZ-jkfit^92^ auxiliary basis set was used for all atoms except iron, for which the def2-universal-jkfit^93^ auxiliary basis set was used.

## 4 Results and Discussion

We aim to understand the stabilization of the spin states in terms of the static and dynamic correlation effects by means of PNOF5, PNOF7s, PNOF7, NOF-MP2, and GNOF calculations. For this purpose, a discussion is given for both the perfect pairing and the extended PNOF approaches, with special attention to the features of the solutions given by each functional.

### 4.1 Perfect Pairing

Here we study the spin-state stability of FeP using the most simple approach for electron-pairing-based NOFs, that is, pairing a single weakly occupied orbital to each strongly occupied orbital in each subspace, namely, the perfect-pairing approach. Table 1 presents the energy values of the singlet, triplet, and quintet states of FeP in its rows, calculated with PNOF5, PNOF7s, PNOF7, NOF-MP2, and GNOF as shown in each column. First, we observe that the energy decreases according to the order PNOF5 > PNOF7s > PNOF7 > GNOF > NOF-MP2 for all spin states, which corresponds to the order of increase of electron correlation in perfect-pairing coupling. In addition, the singlet–triplet (ST) gaps and the quintet-triplet (QT) gaps allow us to check whether the spin state is more stable with respect to the triplet. Positive values indicate that the triplet state is lower in energy, whereas negative values indicate that either the singlet or the quintet state is lower in energy than the triplet state. Overall, PNOF5, PNOF7s, and PNOF7 predict the quintet as the ground state of FeP, which agrees with the multiconfigurational wave functions that include more static correlation, whereas, NOF-MP2 and GNOF afford the expected triplet ground state. The case of GNOF requires a more detailed analysis of the singlet state (vide infra).

**Table 1:** Spin State Energies (Hartree) for FeP Calculated by a Perfect Pairing PNOF5, PNOF7s, PNOF7, NOF-MP2, and GNOF, with its Corresponding Singlet–Triplet Adiabatic Gap (ST), *E*_singlet_–*E*_triplet_, and Quintet–Triplet Adiabatic Gap (QT), *E*_quintet_–*E*_triplet_, in kcal/mol^a^

MUL	PNOF5	PNOF7s	PNOF7	NOF-MP2	GNOF
S	–2245.417	–2245.436	–2245.989	–2248.384	–2247.769
T	–2245.484	–2245.492	–2246.014	–2248.456	–2247.869
ST	42	35	16	45	63
Q	–2245.549	–2245.560	–2246.042	–2248.416	–2247.766
QT	–29	–36	–17	25	65

a The values correspond to calculations using the optimized geometries of ref (74) and the RI approximation.

Take the PNOF5 QT gap as a reference to analyze the results obtained and recall that it considers only static and dynamic intrapair correlation but does not have intersubspace correlation terms that are important for medium and large size systems. These terms are found in PNOF7s and PNOF7 leading to deeper total energy values but predict QT gaps with the wrong sign. It should be noted that PNOF7 predicts a lower QT gap than PNOF7s, a performance associated with the PNOF7 static overcorrelation at the equilibrium structures where the dynamic correlation predominates. In contrast, PNOF7s takes into account the correct amount of static intersubspace correlation; therefore, its energy is in between PNOF5 and PNOF7, but the QT gap prediction is worse due to the lack of the intersubspace dynamic correlation.

NOF-MP2 includes the dynamic correlation taking as reference the Slater determinant formed with the PNOF7s NOs^61^ and predicts the triplet as the ground state, with a QT gap of 25 kcal/mol with the expected sign. This outcome supports the thesis that dynamic correlation is crucial for predicting the triplet as the ground state. In order to obtain GNOF energies, PNOF7s NOs and ONs were used as starting solutions. Since GNOF accounts for static and dynamic correlations, this functional is also capable of predicting the triplet state to be lower in energy than the quintet.

Regarding the singlet state, we must note that all functionals provide a state with a marked multiconfigurational character, as has been reported in previous studies.^76,94^ Remarkably, a ST gap of 17 kcal/mol is achieved by a traditional HF-MP2 calculation that is even lower than the QT gap obtained with the NOF-MP2 method. This result confirms the importance of dynamic correlation and points out the existence of a singlet with a predominant single-reference character.

It should be noted that the total energies of PNOF7s shown in Table 1 are well below the values obtained by Lemke et al.,^40^ namely, −2244.6016 and −2244.6514 for the triplet and quintet states, respectively. The latter are very close to the HF energies and must then correspond to local minima. In contrast, our PNOF7s energies are in better agreement with the results of CAS(44,44).^78^ They are also lower in energy, since they correlate 186 electrons in 184 and 182 orbitals for triplet and quintet states, respectively. Recall that in multiplet states, single-electron subspaces are made up of a single orbital with 2*n*_*g*_ = 1, while electron-paired subspaces are those that follow the perfect pairing.

The M diagnostic of the PNOFs solutions for the spin states of FeP are shown in Table 2. For the triplet and quintet states, PNOF5, PNOF7s, and GNOF provide solutions below 0.1, which indicates that dynamic correlation is the dominant contribution. Note that the solutions of PNOF5 and PNOF7s are close to 0.1, indicating that the static correlation is important despite not being the dominant contribution. In contrast, the results of PNOF7 are strongly dominated by static correlation. It is noteworthy that the singlet states achieved with all functionals present an M-diagnostic value of 1.0, which in the perfect-paring approach directly indicates a diradical character, in agreement with previous reports.^76,94^

**Table 2:** M Diagnostic for the Spin States of FeP Computed with PNOF5, PNOF7s, and PNOF7

MUL	PNOF5	PNOF7s	PNOF7	GNOF
S	1.00	1.00	1.00	1.00
T	0.07	0.07	0.60	0.04
Q	0.07	0.08	0.56	0.04

It has been stated that the NO picture can be used to earn chemical relevant information.^95^ Following this idea, Table 3 presents selected frontier orbitals of each NOF considered in this work for the triplet state. A gradual transformation can be observed from left (PNOF5) to right (GNOF) through an increase in correlation. The main change can be observed in the first row corresponding to the LSNO (equivalent to the HF HOMO), where the effect of the increase of electron correlation is to allow the “d” orbitals of the iron atom to interact with the π orbitals of the porphyrin, as can be seen in PNOF7 and GNOF. A similar effect can be seen in the second and third rows corresponding to the single-electron NOs of the Ω_*I*_ subspace, where the “d” orbital of the iron atoms appears for all PNOFs; however, the NO of GNOFs spreads throughout the molecule. These results are in accordance with the results reported in ref (96), where it is stated that these orbital interactions are the key factor for the correct ordering between the triplet and the quintet states, as achieved by GNOF.

**Table 3:**
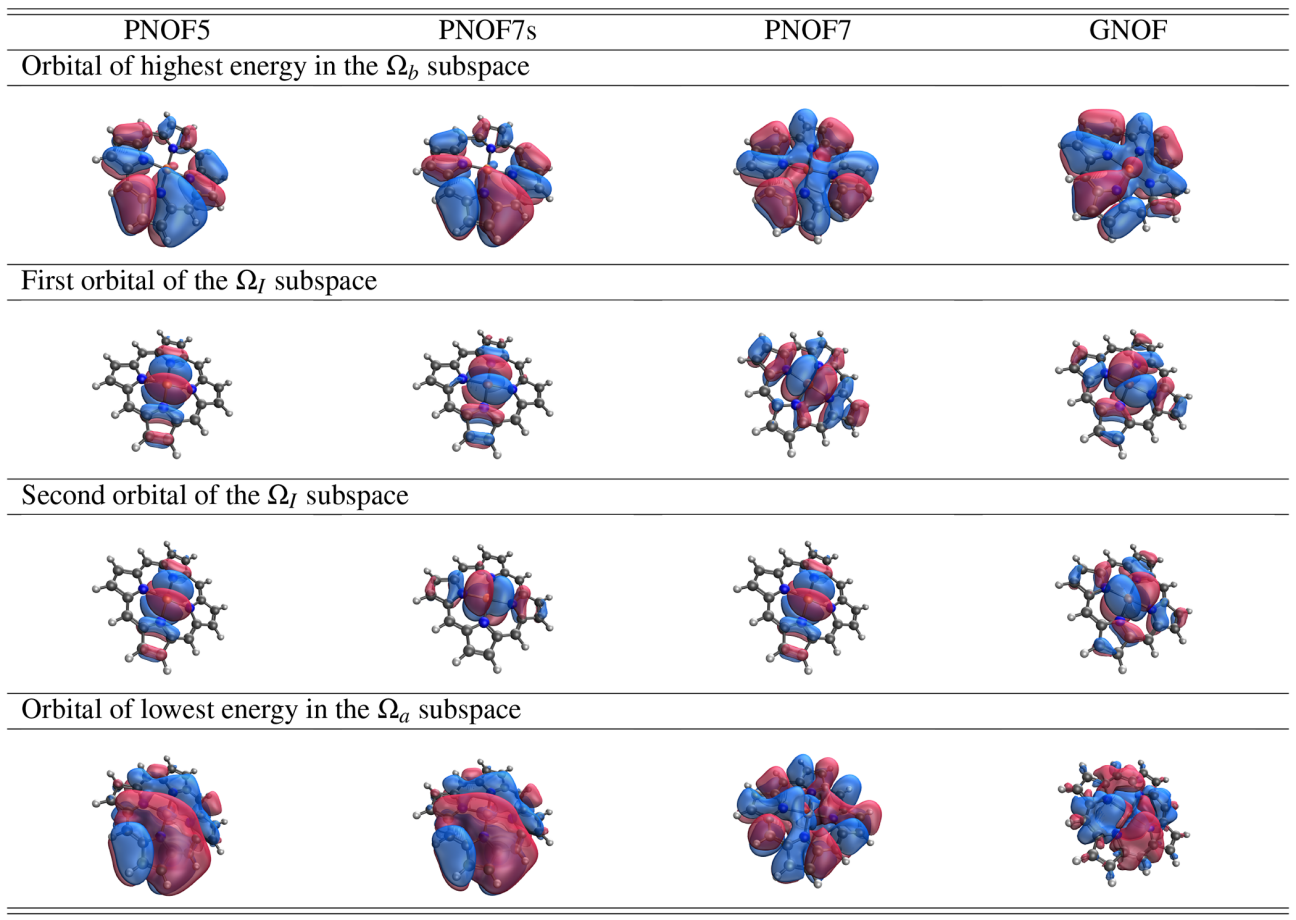
Natural Orbitals of the Triplet State of FeP Computed with PNOF5, PNOF7s, PNOF7, and GNOF

### 4.2 Extended Pairing

In this section, the extended-pairing approach is used to go beyond the results of the previous section. For this purpose, the number of weakly occupied orbitals coupled to each strongly occupied orbital was increased to four; that is, the highest possible coupling with the cc-pVDZ basis set was used. Once again, we used the NOs and ONs obtained with the PNOF7s as inputs to achieve the GNOF solutions. In addition, the full electron repulsion integrals are used.

It can be seen from Table 4 that there is an improvement in the PNOF5 QT gap as the amount of intrapair correlation is increased. As expected, the QT gaps of PNOF7s and GNOF are significantly improved due to an increase in the electron correlation between orbitals that form the single- and paired-electron subspaces, which are not present in the independent pair approximation leading to PNOF5. As noted in the previous section, PNOF7 tends to overestimate the nondynamic electronic correlation between subspaces in the equilibrium region, so these results were not included in the table.

**Table 4:** Spin State Energies (Hartree) for FeP Calculated by Extended Pairing PNOF5, PNOF7s, and GNOF, with Its Corresponding Singlet–Triplet Adiabatic Gap (ST) and Quintet–Triplet Adiabatic Gap (QT), in kcal/mol^a^

MUL	PNOF5	PNOF7s	GNOF
S	–2245.644	–2245.696	–2248.830
T	–2245.742	–2245.748	–2248.855
ST	62	33	16
Q	–2245.766	–2245.776	–2248.784
QT	–15	–16	45

a The optimized geometries of ref (74) are used.

#### 4.2.1 Singlet State with Predominant Dynamic Correlation

As the results of the previous section demonstrate, the inclusion of intersubspace dynamic correlation is crucial for GNOF to favor the intermediate spin state over the low and high spin states. We also noted that the singlet obtained by GNOF from the PNOF7s solution has a marked multiconfigurational character. However, as mentioned above, a traditional MP2 calculation based on the HF reference affords a ST gap that is below the NOF-MP2 result obtained from the reference multiconfigurational PNOF7s singlet. Consequently, we wonder if there is another GNOF singlet state where dynamic correlation predominates. In fact, starting from HF solutions, we have obtained GNOF singlet states with energies of −2247.914 and −2248.918 hartree corresponding to perfect and extended couplings, respectively. Clearly, these energy values favor the singlet with predominant dynamic correlation as the lowest energy state in the GNOF case.

On the other hand, we must be cautious in claiming that this singlet is the state of minimum energy in FeP. If we look more closely at expression 12 that determines the dynamic correlation in GNOF, we can conclude that orbitals with ONs close to half do not contribute to the intersubspace dynamic correlation; that is, it is actually a dynamic correlation term between the electron pairs. Consequently, GNOF does not contain dynamic correlation terms of the single electrons that appear in spin multiplets. This behavior has been observed in other systems, such as molecular oxygen, for which the ST gap is underestimated. In the case of FeP, it could be that GNOF is underestimating the energy of the triplet and quintet states as well as that of the open-shell singlet. It is evident that until we have an improved GNOF that includes the dynamic correlation of those orbitals with total occupancies equal to one (half in the case of the NO), we can only give a partial answer as this study shows.

### 4.3 Computational Times

This work ends with some details related to the time required for the calculations. In particular, the calculations presented in this section were performed in a Julia version of the DoNOF software.^30,48^ Similar codes have been reported^97^ for other electronic structure methods. As noted above, the NOF equations are solved by optimizing the NOs and ONs separately, and these steps together form an outer iteration in the optimization procedure. Our implementation has been tested on an AMD Ryzen 5800 and in two GPUs, the first being a NVIDIA Turing RTX2080 and the second a NVIDIA Tesla V100. For reference, the hardware configurations are the following:

- CPU-only calculations: AMD Ryzen 5800X with 8 cores-16 threads
- GPU calculations:
  - NVIDIA GeForce RTX 2080 with a 8 cores-16 threads AMD Ryzen 5800X CPU
  - NVIDIA Tesla V100 with a 4 cores Intel Xeon Gold 5122 CPU

The integral transformation on the CPU is currently based on Tullio.jl,^98^ while the transformation on the GPU depends mainly on TensorOperations.jl.^99^Table 5 presents the computational times for an outer iteration composed of 30 inner iterations of orbital optimization through the iterative diagonalization algorithm^100^ and the ON optimization using the BFGS algorithm up to |grad| < 10^–4^.

**Table 5:** Computational Times (s) for Calculations Corresponding to the RI Approximation Using a CPU, a NVIDIA GeForce RTX2080 GPU, and a NVIDIA Tesla V100 GPU

	Perfect Pairing (N_g_ = 1)			Extended Pairing (N_g_ = 4)		
Optimization Type	CPU	RTX2080	V100	CPU	RTX2080	V100
NO Optimization	253	10	8	762	22	42
ON Optimization	3	0.2	0.7	109	113	113
Outer Iteration	256	10.2	8.7	871	110	155

A significant improvement in computational time has been achieved in all cases with the GPU implementation, with the cuTENSOR library being a key factor for this success. The integral transformation is the dominating step in both the NO and the ON minimization when the perfect pairing approach is employed; hence the time of an outer iteration is directly benefited when it is performed in a GPU, achieving a speed-up of around 25 times relative to the CPU for the GeFroce RTX2080 GPU and 29 times for the Tesla V100 GPU.

On the other hand, when the CPU is used for the calculations using the extended pairing approach, the NO optimization remains the bottleneck, but the contribution of the ON optimization to the time of an outer iteration increases significantly. In fact, when the GPU is introduced for the integral transformations, the NO optimization time is significantly reduced, as can be seen by going from 762 s on the CPU to 22 s in the RTX2080 hardware configuration, but the ON optimization time remains almost the same; this is the reason the speed-up is reduced to eight times for the extended pairing approach. It is worth noting that the integral transformation is performed only once in the ON optimization; hence, this is not the bottleneck but the calculation of the gradients performed in the CPU. We expect to present further details of this implementation in a future article.

## 5 Conclusions

The PNOFs were used to elucidate the picture of the spin stability order of iron porphyrin. It has been found that NOFs that do not consider a significant amount of dynamic correlation, such as PNOF5, PNOF7s, and PNOF7, favor the quintet as the ground state. In these functionals, the increase of the subspace size improves the results due to the inclusion of dynamic intrapair correlation, but the wrong sign of the quintet–triplet gap remains. On the other hand, methods incorporating significant amounts of dynamic correlation, such as NOF-MP2 and GNOF, achieve the correct prediction for the quintet–triplet gap of FeP and predict the triplet as the ground state if we consider the singlet with multiconfigurational character for GNOF.

Surprisingly, there is another singlet state predicted by GNOF with a predominant dynamic correlation. In principle, this state is the one with the lowest energy, which reinforces the importance of the dynamic correlation in the stability of the iron porphyrin; however, GNOF does not contain dynamic correlation terms for the single electrons that appear in spin multiplets, so we cannot provide a definitive answer at this time, for this finding.

Larger systems, such as FeP with 37 atoms and 186 electrons, have been shown to be affordable for NOFs to handle high levels of correlation. This significantly increases the size of the systems susceptible to multiconfigurational treatment, especially when it comes to graphic processing units, such as those used in this work for the two-electron integral transformation.

In addition, GNOF correlates all electrons in all available orbitals preserving the total spin of multiplet states, which in the case of FeP using a double-ζ basis set implies 186 electrons into 465 orbitals. To the best of our knowledge, such calculations have not been done so far with current wave function-based methods, and they are expected to become a reference calculation.

## References

[ref1] Gilbert T. L. Hohenberg-Kohn theorem for nonlocal external potentials. Phys. Rev. B 1975, 12, 2111–2120. 10.1103/PhysRevB.12.2111.

[ref2] Donnelly R. A.; Parr R. G. Elementary properties of an energy functional of the first-order reduced density matrix. J. Chem. Phys. 1978, 69, 4431–4439. 10.1063/1.436433.

[ref3] Levy M. Universal variational functionals of electron densities, first-order density matrices, and natural spin-orbitals and solution of the v-representability problem. Proc. Natl. Acad. Sci. U.S.A. 1979, 76, 6062–6065. 10.1073/pnas.76.12.6062. 16592733 PMC411802

[ref4] Valone S. M. Consequences of extending 1 matrix energy functionals pure-state representable to all ensemble representable 1 matrices. J. Chem. Phys. 1980, 73, 1344–1349. 10.1063/1.440249.

[ref5] Goedecker S.; Umrigar C. J. In Many-electron densities and reduced density matrices; Cioslowski J., Ed.; Kluwer Academic/Plenum Publishers: New York, 2000; pp 165–181.

[ref6] Piris M. In Reduced-Density-Matrix Mechanics with Application to Many-Electron Atoms and Molecules; Mazziotti D. A., Ed.; John Wiley and Sons: Hoboken, NJ, U.S.A., 2007; Vol. 134; Chapter 14, pp 387–427.

[ref7] Donnelly R. A. On fundamental difference between energy functionals based on first- and second-order density matrices. J. Chem. Phys. 1979, 71, 2874–2879. 10.1063/1.438678.

[ref8] Ludeña E. V.; Torres F. J.; Costa C. Functional N-Representability in 2-Matrix, 1-Matrix, and Density Functional Theories. J. Mod. Phys. 2013, 04, 391–400. 10.4236/jmp.2013.43A055.

[ref9] Piris M. In Many-body approaches at different scales: a tribute to Norman H. March on the occasion of his 90th Birthday; Angilella G. G. N., Amovilli C., Eds.; Springer: New York, 2018; Chapter 22, pp 261–278.

[ref10] Piris M.; Ugalde J. J. M. Perspective on natural orbital functional theory. Int. J. Quantum Chem. 2014, 114, 1169–1175. 10.1002/qua.24663.

[ref11] Pernal K.; Giesbertz K. J. H. Reduced Density Matrix Functional Theory (RDMFT) and Linear Response Time-Dependent RDMFT (TD-RDMFT). Top Curr. Chem. 2015, 368, 125–184. 10.1007/128_2015_624. 25971917

[ref12] Schade R.; Kamil E.; Blöchl P. Reduced density-matrix functionals from many-particle theory. Eur. Phys. J. Spec. Top. 2017, 226, 2677–2692. 10.1140/epjst/e2017-70046-0.

[ref13] Mitxelena I.; Piris M.; Ugalde J. M. In State of The Art of Molecular Electronic Structure Computations: Correlation Methods, Basis Sets and More; Hoggan P., Ancarani U., Eds.; Advances in Quantum Chemistry; Academic Press: 2019; Vol. 79, Chapter 7, pp 155–177.

[ref14] Piris M. Natural orbital functional for multiplets. Phys. Rev. A 2019, 100, 032508 10.1103/PhysRevA.100.032508.

[ref15] Benavides-Riveros C. L.; Marques M. A. On the time evolution of fermionic occupation numbers. J. Chem. Phys. 2019, 151, 044112 10.1063/1.5109009. 31370532

[ref16] Cioslowski J.; Mihalka Z. E.; Szabados A. Bilinear Constraints Upon the Correlation Contribution to the Electron-Electron Repulsion Energy as a Functional of the One-Electron Reduced Density Matrix. J. Chem. Theory Comput. 2019, 15, 4862–4872. 10.1021/acs.jctc.9b00443. 31294976

[ref17] Giesbertz K. J.; Ruggenthaler M. One-body reduced density-matrix functional theory in finite basis sets at elevated temperatures. Phys. Rep. 2019, 806, 1–47. 10.1016/j.physrep.2019.01.010.

[ref18] Gritsenko O. V.; Pernal K. Approximating one-matrix functionals without generalized Pauli constraints. Phys. Rev. A 2019, 100, 012509 10.1103/PhysRevA.100.012509.

[ref19] Lopez X.; Piris M. Performance of the NOF-MP2 method in hydrogen abstraction reactions. Theor. Chem. Acc. 2019, 138, 89 10.1007/s00214-019-2475-5.

[ref20] Quintero-Monsebaiz R.; Mitxelena I.; Rodríguez-Mayorga M.; Vela A.; Piris M. Natural orbital functional for spin-polarized periodic systems. J. Phys.: Condens. Matter 2019, 31, 165501–8. 10.1088/1361-648X/ab0170. 30673638

[ref21] Schilling C.; Schilling R. Diverging Exchange Force and Form of the Exact Density Matrix Functional. Phys. Rev. Lett. 2019, 122, 013001–7. 10.1103/PhysRevLett.122.013001. 31012728

[ref22] Schmidt J.; Benavides-Riveros C. L.; Marques M. A. L. Reduced Density Matrix Functional Theory for Superconductors. Phys. Rev. B 2019, 99, 224502 10.1103/PhysRevB.99.224502.

[ref23] Buchholz F.; Theophilou I.; Nielsen S. E. B.; Ruggenthaler M.; Rubio A. Reduced Density-Matrix Approach to Strong Matter-Photon Interaction. ACS Photonics 2019, 6, 2694–2711. 10.1021/acsphotonics.9b00648. 31788499 PMC6875895

[ref24] Benavides-Riveros C. L.; Wolff J.; Marques M. A.; Schilling C. Reduced Density Matrix Functional Theory for Bosons. Phys. Rev. Lett. 2020, 124, 180603 10.1103/PhysRevLett.124.180603. 32441966

[ref25] Giesbertz K. J. Implications of the unitary invariance and symmetry restrictions on the development of proper approximate one-body reduced-density-matrix functionals. Phys. Rev. A 2020, 102, 052814 10.1103/PhysRevA.102.052814.

[ref26] Cioslowski J. The One-Electron Reduced Density Matrix Functional Theory of Spin-Polarized Systems. J. Chem. Theory Comput. 2020, 16, 1578–1585. 10.1021/acs.jctc.9b01155. 31904973

[ref27] Mitxelena I.; Piris M. An efficient method for strongly correlated electrons in one dimension. J. Phys.: Condens. Matter 2020, 32, 17LT01 10.1088/1361-648X/ab6d11. 31952058

[ref28] Mitxelena I.; Piris M. An efficient method for strongly correlated electrons in two-dimensions. J. Chem. Phys. 2020, 152, 064108 10.1063/1.5140985. 32061239

[ref29] Mitxelena I.; Piris M. Analytic gradients for spin multiplets in natural orbital functional theory. J. Chem. Phys. 2020, 153, 044101 10.1063/5.0012897. 32752719

[ref30] Lew-Yee J. F. H.; Piris M.; del Campo J. M. Resolution of the identity approximation applied to PNOF correlation calculations. J. Chem. Phys. 2021, 154, 064102 10.1063/5.0036404. 33588540

[ref31] Mercero J. M.; Ugalde J. M.; Piris M. Chemical reactivity studies by the natural orbital functional second-order Møller–Plesset (NOF-MP2) method: water dehydrogenation by the scandium cation. Theor. Chem. Acc. 2021, 140, 74 10.1007/s00214-021-02775-4.

[ref32] Quintero-Monsebaiz R.; Perea-Ramirez L. I.; Piris M.; Vela A. Spectroscopic properties of open shell diatomic molecules using Piris Natural Orbital Functionals. Phys. Chem. Chem. Phys. 2021, 23, 2953–2963. 10.1039/D0CP05430E. 33480904

[ref33] Schilling C.; Pittalis S. Ensemble Reduced Density Matrix Functional Theory for Excited States and Hierarchical Generalization of Pauli’s Exclusion Principle. Phys. Rev. Lett. 2021, 127, 023001 10.1103/PhysRevLett.127.023001. 34296916

[ref34] Liebert J.; Schilling C. Functional theory for Bose–Einstein condensates. Phys. Rev. Res. 2021, 3, 013282 10.1103/PhysRevResearch.3.013282.

[ref35] Wang Y.; Knowles P. J.; Wang J. Information entropy as a measure of the correlation energy associated with the cumulant. Phys. Rev. A 2021, 103, 062808 10.1103/PhysRevA.103.062808.

[ref36] Yao Y.-F.; Fang W.-H.; Su N. Q. Handling Ensemble N-Representability Constraint in Explicit-by-Implicit Manner. J. Phys. Chem. Lett. 2021, 12, 6788–6793. 10.1021/acs.jpclett.1c01835. 34270236

[ref37] Gibney D.; Boyn J. N.; Mazziotti D. A. Toward a Resolution of the Static Correlation Problem in Density Functional Theory from Semidefinite Programming. J. Phys. Chem. Lett. 2021, 12, 385–391. 10.1021/acs.jpclett.0c03371. 33356286

[ref38] Piris M. Global Natural Orbital Functional: Towards the Complete Description of the Electron Correlation. Phys. Rev. Lett. 2021, 127, 233001 10.1103/PhysRevLett.127.233001. 34936779

[ref39] Di Sabatino S.; Koskelo J.; Berger J. A.; Romaniello P. Introducing screening in one-body density matrix functionals: impact on the Extended Koopmans’ Theorem’s charged excitations of model systems. Phys. Rev. B 2022, 105, 235123 10.1103/PhysRevB.105.235123.

[ref40] Lemke Y.; Kussmann J.; Ochsenfeld C. Efficient Integral-Direct Methods for Self-Consistent Reduced Density Matrix Functional Theory Calculations on Central and Graphics Processing Units. J. Chem. Theory Comput. 2022, 18, 4229–4244. 10.1021/acs.jctc.2c00231. 35818791

[ref41] Mitxelena I.; Piris M. Benchmarking GNOF against FCI in challenging systems in one, two, and three dimensions. J. Chem. Phys. 2022, 156, 214102 10.1063/5.0092611. 35676141

[ref42] Liebert J.; Castillo F.; Labbé J.-P.; Schilling C. Foundation of one-particle reduced density matrix functional theory for excited states. J. Chem. Theory Comput. 2022, 18, 124–140. 10.1021/acs.jctc.1c00561. 34931830

[ref43] Wang J.; Baerends E. J. Self-Consistent-Field Method for Correlated Many-Electron Systems with an Entropic Cumulant Energy. Phys. Rev. Lett. 2022, 128, 013001 10.1103/PhysRevLett.128.013001. 35061466

[ref44] Ding L.; Liebert J.; Schilling C. Comment on ”Self-Consistent-Field Method for Correlated Many-Electron Systems with an Entropic Cumulant Energy. arXiv, 2022, 2202.05532. 10.1103/PhysRevLett.128.013001 35061466

[ref45] Rodríguez-Mayorga M.; Giesbertz K. J. H.; Visscher L. Relativistic reduced density matrix functional theory. SciPost Chem. 2022, 1, 004 10.21468/SciPostChem.1.2.004.

[ref46] Senjean B.; Yalouz S.; Nakatani N.; Fromager E. Reduced density matrix functional theory from an ab initio seniority-zero wave function: Exact and approximate formulations along an adiabatic connection path. Phys. Rev. A 2022, 106, 032203 10.1103/PhysRevA.106.032203.

[ref47] Lew-Yee J. F. H.; Del Campo J. M. Charge delocalization error in Piris natural orbital functionals. J. Chem. Phys. 2022, 157, 104113 10.1063/5.0102310. 36109213

[ref48] Piris M.; Mitxelena I. DoNOF: an open-source implementation of natural-orbital-functional-based methods for quantum chemistry. Comput. Phys. Commun. 2021, 259, 107651–14. 10.1016/j.cpc.2020.107651.

[ref49] Mitxelena I.; Piris M.; Rodriguez-Mayorga M. On the performance of natural orbital functional approximations in the Hubbard model. J. Phys.: Condens. Matter 2017, 29, 425602 10.1088/1361-648X/aa80ca. 28722686

[ref50] Piris M. In Theoretical and Quantum Chemistry at the Dawn of the 21st Century; Carbó-Dorca R., Chakraborty T., Eds.; Innovations in Computational Chemistry; Apple Academic Press: 2018; Chapter 22, pp 593–620.

[ref51] Piris M.; Lopez X.; Ruipérez F.; Matxain J. M.; Ugalde J. M. A natural orbital functional for multiconfigurational states. J. Chem. Phys. 2011, 134, 164102 10.1063/1.3582792. 21528945

[ref52] Piris M.; Matxain J. M.; Lopez X. The intrapair electron correlation in natural orbital functional theory. J. Chem. Phys. 2013, 139, 234109–9. 10.1063/1.4844075. 24359354

[ref53] Piris M. Interacting pairs in natural orbital functional theory. J. Chem. Phys. 2014, 141, 044107 10.1063/1.4890653. 25084881

[ref54] Piris M. Global Method for Electron Correlation. Phys. Rev. Lett. 2017, 119, 063002–5. 10.1103/PhysRevLett.119.063002. 28949623

[ref55] Mitxelena I.; Rodríguez-Mayorga M.; Piris M. Phase Dilemma in Natural Orbital Functional Theory from the N-representability Perspective. Eur. Phys. J. B 2018, 91, 109 10.1140/epjb/e2018-90078-8.

[ref56] Piris M.; Lopez X.; Ugalde J. J. M. J. The Bond Order of C 2 from an Strictly N-Representable Natural Orbital Energy Functional Perspective. Chem. - A Eur. J. 2016, 22, 4109 10.1002/chem.201504491. 26822104

[ref57] Matxain J. M.; Piris M.; Ruipérez F.; Lopez X.; Ugalde J. M. Homolytic molecular dissociation in natural orbital functional theory. Phys. Chem. Chem. Phys. 2011, 13, 20129–20135. 10.1039/c1cp21696a. 21904734

[ref58] Ruipérez F.; Piris M.; Ugalde J. M.; Matxain J. M. The natural orbital functional theory of the bonding in Cr(2), Mo(2) and W(2). Phys. Chem. Chem. Phys. 2013, 15, 2055–2062. 10.1039/C2CP43559D. 23262452

[ref59] Piris M. Interpair electron correlation by second-order perturbative corrections to PNOF5. J. Chem. Phys. 2013, 139, 064111–7. 10.1063/1.4817946. 23947847

[ref60] Piris M.; Ruipérez F.; Matxain J. Assessment of the second-order perturbative corrections to PNOF5. Mol. Phys. 2014, 112, 1–8. 10.1080/00268976.2013.854933.

[ref61] Piris M. Dynamic electron-correlation energy in the natural-orbital-functional second-order-Møller-Plesset method from the orbital-invariant perturbation theory. Phys. Rev. A 2018, 98, 022504–6. 10.1103/PhysRevA.98.022504.

[ref62] Rodríguez-Mayorga M.; Mitxelena I.; Bruneval F.; Piris M. Coupling Natural Orbital Functional Theory and Many-Body Perturbation Theory by Using Nondynamically Correlated Canonical Orbitals. J. Chem. Theory Comput. 2021, 17, 7562–7574. 10.1021/acs.jctc.1c00858. 34806362

[ref63] Lever A. B. P.; Gray H. B. Iron Porphyrins Part 3; Wiley-VCH: 1989; p 322.

[ref64] Ugalde J. M.; Dunietz B.; Dreuw A.; Head-Gordon M.; Boyd R. J. The spin dependence of the spatial size of Fe(II) and of the structure of Fe(II)-porphyrins. J. Phys. Chem. A 2004, 108, 4653–4657. 10.1021/jp0489119.

[ref65] Perutz M. F.; Wilkinson A. J.; Paoli M.; Dodson G. G. The stereochemical mechanism of the cooperative effects in hemoglobin revisited. Annu. ReV. Biophys. Biomol. Struct. 1998, 27, 1 10.1146/annurev.biophys.27.1.1. 9646860

[ref66] Dayan F.; Dayan E. Porphyrins: One ring in the colors of life. Am. Sci. 2011, 99, 236 10.1511/2011.90.236.

[ref67] Obara S.; Kashiwagi H. Ab initio MO studies of electronic states and Mössbauer spectra of high-, intermediate-, and low-spin Fe(II)-porphyrin complexes. J. Chem. Phys. 1982, 77, 3155–3165. 10.1063/1.444239.

[ref68] Sontum S. F.; Case D. A.; Karplus M. Xα multiple scattering calculations on iron(II) porphine. J. Chem. Phys. 1983, 79, 2881–2892. 10.1063/1.446110.

[ref69] Rohmer M.-M. Electronic ground state of iron(II)porphyrin. Ab initio SCF and CI calculations and computed electron deformation densities. Chem. Phys. Lett. 1985, 116, 44–49. 10.1016/0009-2614(85)80122-6.

[ref70] Rawlings D. C.; Gouterman M.; Davidson E. R.; Feller D. Theoretical investigations of the electronic states of porphyrins. III. Low-lying electronic states of porphinatoiron(II). Int. J. Quantum Chem. 1985, 28, 773–796. 10.1002/qua.560280611.

[ref71] Choe Y. K.; Hashimoto T.; Nakano H.; Hirao K. Theoretical study of the electronic ground state of iron(II) porphine. Chem. Phys. Lett. 1998, 295, 380–388. 10.1016/S0009-2614(98)00986-5.

[ref72] Choe Y. K.; Nakajima T.; Hirao K.; Lindh R. Theoretical study of the electronic ground state of iron(II) porphine. II. J. Chem. Phys. 1999, 111, 3837–3845. 10.1063/1.479687.

[ref73] Liao M.-S.; Scheiner S. Electronic structure and bonding in metal porphyrins, metal = Fe, Co, Ni, Cu, Zn. J. Chem. Phys. 2002, 117, 205–219. 10.1063/1.1480872.

[ref74] Groenhof A. R.; Swart M.; Ehlers A. W.; Lammertsma K. Electronic ground states of iron porphyrin and of the first species in the catalytic reaction cycle of cytochrome P450s. J. Phys. Chem. A 2005, 109, 3411–3417. 10.1021/jp0441442. 16833677

[ref75] Radoń M. Spin-State Energetics of Heme-Related Models from DFT and Coupled Cluster Calculations. J. Chem. Theory Comput. 2014, 10, 2306–2321. 10.1021/ct500103h. 26580751

[ref76] Lee J.; Malone F. D.; Morales M. A. Utilizing Essential Symmetry Breaking in Auxiliary-Field Quantum Monte Carlo: Application to the Spin Gaps of the C36 Fullerene and an Iron Porphyrin Model Complex. J. Chem. Theory Comput. 2020, 16, 3019–3027. 10.1021/acs.jctc.0c00055. 32283932

[ref77] Li Manni G.; Smart S. D.; Alavi A. Combining the Complete Active Space Self-Consistent Field method and the Full Configuration Interaction Quantum Monte Carlo within a Super-CI framework, with application to challenging metal-porphyrins. J. Chem. Theory Comput. 2016, 12, 1245–1258. 10.1021/acs.jctc.5b01190. 26808894

[ref78] Smith J. E.; Mussard B.; Holmes A. A.; Sharma S. Cheap and Near Exact CASSCF with Large Active Spaces. J. Chem. Theory Comput. 2017, 13, 5468–5478. 10.1021/acs.jctc.7b00900. 28968097

[ref79] Pierloot K.; Phung Q. M.; Domingo A. Spin State Energetics in First-Row Transition Metal Complexes: Contribution of (3s3p) Correlation and Its Description by Second-Order Perturbation Theory. J. Chem. Theory Comput. 2017, 13, 537–553. 10.1021/acs.jctc.6b01005. 28005368

[ref80] Zhou C.; Gagliardi L.; Truhlar D. G. Multiconfiguration Pair-Density Functional Theory for Iron Porphyrin with CAS, RAS, and DMRG Active Spaces. J. Phys. Chem. A 2019, 123, 3389–3394. 10.1021/acs.jpca.8b12479. 30763100

[ref81] Weser O.; Guther K.; Ghanem K.; Li Manni G. Stochastic Generalized Active Space Self-Consistent Field: Theory and Application. J. Chem. Theory Comput. 2022, 18, 251–272. 10.1021/acs.jctc.1c00936. 34898215 PMC8757470

[ref82] Antalík A.; Nachtigallová D.; Lo R.; Matoušek M.; Lang J.; Legeza Ö.; Pittner J.; Hobza P.; Veis L. Ground state of the Fe(ii)-porphyrin model system corresponds to quintet: a DFT and DMRG-based tailored CC study. Phys. Chem. Chem. Phys. 2020, 22, 17033–17037. 10.1039/D0CP03086D. 32716452

[ref83] Beran P.; Matoušek M.; Hapka M.; Pernal K.; Veis L. Density Matrix Renormalization Group with Dynamical Correlation via Adiabatic Connection. J. Chem. Theory Comput. 2021, 17, 7575–7585. 10.1021/acs.jctc.1c00896. 34762423

[ref84] Swart M.; Groenhof A. R.; Ehlers A. W.; Lammertsma K. Validation of exchange-correlation functionals for spin states of iron complexes. J. Phys. Chem. A 2004, 108, 5479–5483. 10.1021/jp049043i.

[ref85] Berryman V. E. J.; Boyd R. J.; Johnson E. R. Balancing Exchange Mixing in Density-Functional Approximations for Iron Porphyrin. J. Chem. Theory Comput. 2015, 11, 3022–3028. 10.1021/acs.jctc.5b00203. 26575739

[ref86] Stroscio G. D.; Zhou C.; Truhlar D. G.; Gagliardi L. Multiconfiguration Pair-Density Functional Theory Calculations of Iron(II) Porphyrin: Effects of Hybrid Pair-Density Functionals and Expanded RAS and DMRG Active Spaces on Spin-State Orderings. J. Phys. Chem. A 2022, 126, 3957–3963. 10.1021/acs.jpca.2c02347. 35674705

[ref87] Tishchenko O.; Zheng J.; Truhlar D. G. Multireference model chemistries for thermochemical kinetics. J. Chem. Theory Comput. 2008, 4, 1208–1219. 10.1021/ct800077r. 26631697

[ref88] Guo Y.; Zhang N.; Lei Y.; Liu W. iCISCF: An Iterative Configuration Interaction-Based Multiconfigurational Self-Consistent Field Theory for Large Active Spaces. J. Chem. Theory Comput. 2021, 17, 7545–7561. 10.1021/acs.jctc.1c00781. 34757746

[ref89] Dunning T. H.; Dunning T. H. Jr. Gaussian basis sets for use in correlated molecular calculations. I. The atoms boron through neon and hydrogen. J. Chem. Phys. 1989, 90, 1007–1023. 10.1063/1.456153.

[ref90] Balabanov N. B.; Peterson K. A. Systematically convergent basis sets for transition metals. I. All-electron correlation consistent basis sets for the 3d elements Sc-Zn. J. Chem. Phys. 2005, 123, 064107–15. 10.1063/1.1998907. 16122300

[ref91] Lew-Yee J. F. H.; Piris M.; Del Campo J. M. Resolution of the identity approximation applied to PNOF correlation calculations. J. Chem. Phys. 2021, 154, 064102 10.1063/5.0036404. 33588540

[ref92] Weigend F. A fully direct RI-HF algorithm: Implementation, optimized auxiliary basis sets, demonstration of accuracy and efficiency. Phys. Chem. Chem. Phys. 2002, 4, 4285–4291. 10.1039/b204199p.

[ref93] Weigend F. Hartree-Fock exchange fitting basis sets for H to Rn. J. Comput. Chem. 2008, 29, 167–175. 10.1002/jcc.20702. 17568435

[ref94] Rovira C.; Kunc K.; Hutter J.; Ballone P.; Parrinello M. Equilibrium Geometries and Electronic Structure of Iron-Porphyrin Complexes: A Density Functional Study. J. Phys. Chem. A 1997, 101, 8914–8925. 10.1021/jp9722115.

[ref95] Piris M.; Matxain J. M.; Lopez X.; Ugalde J. M. The one-electron picture in the Piris natural orbital functional 5 (PNOF5). Theor. Chem. Acc. 2013, 132, 1298 10.1007/s00214-012-1298-4.

[ref96] Manni G. L.; Alavi A. Understanding the Mechanism Stabilizing Intermediate Spin States in Fe(II)-Porphyrin. J. Phys. Chem. A 2018, 122, 4935–4947. 10.1021/acs.jpca.7b12710. 29595978

[ref97] Aroeira G. J. R.; Davis M. M.; Turney J. M.; Schaefer H. F. 3rd Fermi.jl: A Modern Design for Quantum Chemistry. J. Chem. Theory Comput. 2022, 18, 677–686. 10.1021/acs.jctc.1c00719. 34978451

[ref98] Abbott M.; Aluthge D.; N3N; Schaub S.; Elrod C.; Lucibello C.; Chen J. mcabbott/Tullio.jl, v0.3.5.2022; https://github.com/mcabbott/Tullio.jl.

[ref99] Jutho; getzdan; Lyon S.; Protter M.; Marcus P. S.; Leo; Garrison J.; Otto F.; Saba E.; Iouchtchenko D.; Privett A.; Morley A. TensorOperations.jl, v1.1.0. 2019; https://github.com/Jutho/TensorOperations.jl.

[ref100] Piris M.; Ugalde J. M. Iterative Diagonalization for Orbital Optimization in Natural Orbital Functional Theory. J. Comput. Chem. 2009, 30, 2078–2086. 10.1002/jcc.21225. 19219918

